# Structural Masquerade of *Plesiomonas shigelloides* Strain CNCTC 78/89 *O-*Antigen—High-Resolution Magic Angle Spinning NMR Reveals the Modified d-galactan I of *Klebsiella pneumoniae*

**DOI:** 10.3390/ijms18122572

**Published:** 2017-11-29

**Authors:** Karolina Ucieklak, Sabina Koj, Damian Pawelczyk, Tomasz Niedziela

**Affiliations:** Hirszfeld Institute of Immunology and Experimental Therapy, Polish Academy of Sciences, 53-114 Wroclaw, Poland; karolina.ucieklak@iitd.pan.wroc.pl (K.U.); sabina.koj@iitd.pan.wroc.pl (S.K.); damianpawelczyk@yahoo.pl (D.P.)

**Keywords:** *Plesiomonas shigelloides*, *O-*antigen, lipopolysaccharide, *O-*acetylation, d-galactan I, HR-MAS, NMR spectroscopy

## Abstract

The high-resolution magic angle spinning nuclear magnetic resonance spectroscopy (HR-MAS NMR) analysis of *Plesiomonas shigelloides* 78/89 lipopolysaccharide directly on bacteria revealed the characteristic structural features of the *O*-acetylated polysaccharide in the NMR spectra. The *O*-antigen profiles were unique, yet the pattern of signals in the, spectra along with their ^1^H,^13^C chemical shift values, resembled these of d-galactan I of *Klebsiella pneumoniae*. The isolated *O-*specific polysaccharide (O-PS) of *P. shigelloides* strain CNCTC 78/89 was investigated by ^1^H and ^13^C NMR spectroscopy, mass *spectrometry* and chemical methods. The analyses demonstrated that the *P. shigelloides* 78/89 *O-*PS is composed of →3)-α-d-Gal*p*-(1→3)-β-d-Gal*f*2OAc-(1→ disaccharide repeating units. The *O-*acetylation was incomplete and resulted in a microheterogeneity of the *O-*antigen. This *O-*acetylation generates additional antigenic determinants within the *O-*antigen, forms a new chemotype, and contributes to the epitopes recognized by the *O-*serotype specific antibodies. The serological cross-reactivities further confirmed the inter-specific structural similarity of these *O-*antigens.

## 1. Introduction

*Plesiomonas shigelloides* is a facultative anaerobic Gram-negative flagellated, rod-shaped bacterium belonging to the *Enterobacteriaceae* family [1]. It is widely distributed in nature, but predominantly isolated from aquatic environments and animals [2]. These bacteria are not part of the natural human microflora. Human infections with *P. shigelloides* are generally related to visiting countries with low sanitary standards [3,4], drinking unpurified water, and eating uncooked shellfish [5,6]. These bacteria are potent inducers of an invasive shigellosis-like disease [7], gastroenteritis [8], and diarrheal disease [9]. Although the pathogenicity of *P. shigelloides* is not entirely understood, lipopolysaccharide (LPS) is considered the main virulence factor. LPS is a major component of the outer leaflet of the external membrane of Gram-negative bacteria. These are amphiphilic molecules isolated from smooth bacterial strains (S-LPS). Structurally, they can be divided into three distinct regions: lipid A, core oligosaccharide, and *O-*specific polysaccharide. These segments are important for the biological activity [10] and take part in host–bacterium interactions [11]. The hydrophobic lipid A constitutes the most conserved part of LPS, yet lipid A structures and the endotoxic activities that they imply vary substantially between different species of Gram-negative bacteria [12]. The core oligosaccharides (OS) have structures that are generally conserved within bacterial species. The *O-*specific polysaccharide (*O*-antigen, *O-*specific chain) determines bacterial *O-*serotype and constitutes a fingerprint of bacteria [13]. The high structural diversity of the *O-*specific polysaccharides provides the serological distinction between bacterial strains. The variability of *O-*antigen structures is one of the strategies used by bacteria to avoid recognition by host organisms and to hamper the host’s defenses. Despite this variability, serological cross-reactivities between various species occur, indicating the presence of common structural epitopes within *O-*antigens. To date only several LPS structures out of 102 *O-*serotypes of *P. shigelloides* [14] have been analyzed and reported [15,16,17,18,19,20,21,22,23,24,25]. All these studies exposed several characteristic features of *P. shigelloides* LPSs, i.e., the lack of phosphate groups, the presence of uronic acid residues in the core oligosaccharides, and the unusual hydrophobicity of the *O-*specific polysaccharides [22]. Most of the polysaccharides are unique to the *Plesiomonas* species and distinguish them from other members of the *Enterobacteriaceae* family. However, some *O-*antigens of *P. shigelloides* have shown cross-reactivity with antisera directed against LPS of *Shigella* spp. The structure of *P. shigelloides* serotype O17 was found to be identical to the *Shigella sonnei* phase I *O-*chain [19,26,27]. Two other strains of *P. shigelloides* share a type-specific antigen with *S. flexneri* and *S. dysenteriae* [15,28]. The structural element α-l-Rha*p*(1→2)-α-l-Rha*p* described in *S. flexneri* serotype 6 is shared with *P. shigelloides* and *Klebsiella pneumoniae O-*antigens [29]. In some aspects, this cross-reactivity of antibodies against the *O-*antigens is desirable as it can provide broad protection against heterologous bacteria.

We have now identified a new *O-*antigen of *P. shigelloides*, structurally similar to this of *K. pneumoniae* strain Kp20. Herein, we present the chemotyping of the *O-*antigen of *P. shigelloides* strain CNCTC 78/89 by high-resolution magic angle spinning (HR-MAS) nuclear magnetic resonance (NMR) spectroscopy in situ alongside the structural analysis of the isolated *O-*specific polysaccharide, which supports this preliminary observations. The shared epitopes of *Klebsiella* and *Plesiomonas O-*antigens, responsible for their serological cross-reactivities, have also been determined.

## 2. Results

### 2.1. HR-MAS NMR Analysis of P. shigelloides 78/89 Bacteria and LPS

**Figure 1:**
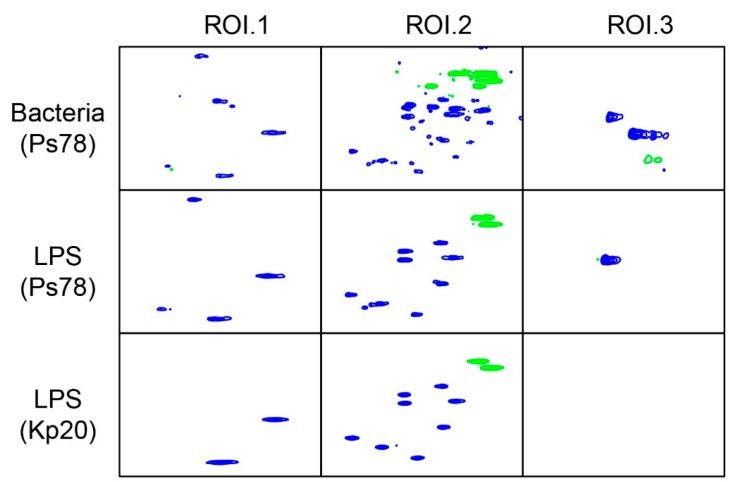
Regions of interest (ROI) extracted from the high-resolution magic angle spinning (HR-MAS) HSQC-DEPT NMR spectra of the *O-*antigens of *P. shigelloides* strain CNCTC 78/89 (Ps78) bacteria and LPS compared to these of *K. pneumoniae* strain Kp20 LPS. The regions were selected directly from the SPARKY processed spectra using the rNMR software. The compared regions and their chemical shift ranges include: anomeric signals (ROI.1, δ_H_ 5.45–4.96 ppm, δ_C_ 112.6–79.5 ppm), the ring resonances (ROI.2, δ_H_ 4.57–3.51 ppm, δ_C_ 88.9–54.4 ppm) and acetyl-group resonances (ROI.3, δ_H_ 2.36–1.81 ppm, δ_C_ 27.6–13.5 ppm). The HSQC-DEPT NMR spectra of bacteria (~4 mg dry mass) and LPS (~3 mg) suspensions in ^2^H_2_O (total volume of ~30 µL in the Bruker Kel-F inserts) were obtained using a Bruker 4 mm HR-MAS probe on an Avance III 600 MHz spectrometer. The experiments were carried out using a ZrO_2_ rotor at a spin rate of 4 kHz at 27 °C (the actual temperature of the bearing gas).

The *O-*antigens of *P. shigelloides* 78/89 were initially investigated by HR-MAS NMR spectroscopy as the technique allows for the direct identification of the flexible *O-*antigen molecules on the bacterial cells in situ. The screening of the whole bacteria of *P. shigelloides* 78/89 using HR-MAS NMR technique provided data on the *O-*PS spectral pattern of this strain. The HR-MAS NMR spectra of *P. shigelloides* 78/89 bacteria were complex and contained signals for anomeric and ring protons, as well as resonances of other surface molecules and metabolites (Figure S1). The observed ^1^H resonances and ^1^H,^13^C-correlations in the HR-MAS NMR spectra of bacteria were further complemented by the HR-MAS NMR analysis of the isolated LPS. Both sets of data revealed distinct structural features of the *O-*acetylated polysaccharide in the *O-*antigen of *P. shigelloides* 78/89. The spectra of the isolated *P. shigelloides* 78/89 LPS contained main signals for three anomeric protons, resonances of the ring protons and a distinct signal in the region of acetyl groups (δ_H_ 2.11 ppm). The observed pattern of resonances was compared with the *O-*antigen structural data available in our laboratory, including LPSs of various *Klebsiella strains,* and the data published previously. This *O-*antigen profile was unique, however, in that a subset of signals in the 1D and HSQC-DEPT spectra as well as the ^1^H, ^13^C chemical shift values were similar to these of *K. pneumoniae* strain Kp20 LPS (Figure 1). To unscramble this similarity, the LPS of *P. shigelloides* 78/89 was subjected to further structural analyses.

### 2.2. Isolation of LPS and O-Antigen Fractions

The LPS of *P. shigelloides* CNCTC 78/89 was extracted from bacterial mass by the hot phenol/water method and purified by ultracentrifugation. The heteropolysaccharide components were released by mild acid hydrolysis of the LPS and isolated by gel filtration on Bio-Gel P-10, yielding four main fractions. The fractions were analyzed by matrix-assisted laser desorption ionization time-of-flight (MALDI-TOF) mass spectrometry and NMR spectroscopy, and identified as the *O-*specific polysaccharide fraction (PSI), fraction composed of short *O-*specific chains substituted by core oligosaccharide (OSII) and the core oligosaccharide (OSIII and OSIV) (Figure S2).

**Figure 2:**
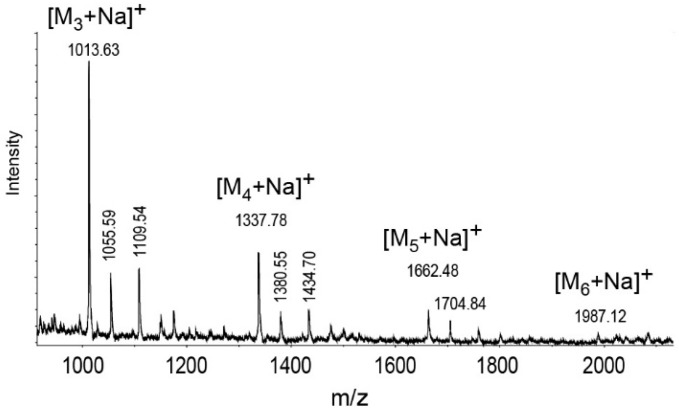
Matrix-assisted laser desorption ionization time-of-flight (MALDI-TOF) mass spectrum of the partially hydrolyzed polysaccharide fraction of *Plesiomonas shigelloides* 78/89 LPS. The MALDI-TOF mass spectrum was obtained in a positive linear mode. 2,5-Dihydroxybenzoic was used as matrix. M_3_, M_4_, M_5_, and M_6_ represent the mass of the respective number of repeating units.

As the attempts to obtain the MALDI-TOF spectra of the intact PSI failed, the mass of the repeating unit of *P. shigelloides* strain 78/89 has been deduced from the analysis of the partially hydrolyzed PSI fraction. The PSI fraction was subjected to a partial acid hydrolysis with 0.5 M TFA. The MALDI-TOF mass spectrum (Figure 2) showed the clusters of ions corresponding to oligosaccharide fragments consisting of 3 up to 6 repeating units. The main signal at *m*/*z* 1013.63 Da corresponded to an oligosaccharide fragment comprising three repeating units and it was accompanied by a minor signal (*m*/*z* 1055.59) of the *O-*acetyled variant of the oligosaccharide. The observed mass differences indicated the disaccharide (Δ 324 Da) and the *O-*acetylated disaccharide (Δ 366 Da) *O-*repeats.

The combined NMR and MS data of the more abundant core oligosaccharide fraction (OSIV) indicated the presence of 11 sugar residues, together having a monoisotopic mass of 2015.65 Da. The chemical shift values of the spin systems of the OSIV were compared with these for other core types of *P. shigelloides* described to date. The acquired NMR data of the OSIV appeared very much like the previously published core oligosaccharide structure of *P. shigelloides* O33:H3 (strain CNCTC 34/89) [30], with two noticeable differences. The -4)-α-Gal*p*NAc-(1-residue in the core of *P. shigelloides* 78/89 is not *O-*acetylated. The disaccharide element in the outer core is built of -4)-α-Gal*p*NAc-(1→6)-α-Glc*p*N-(1-, and there is no heterogeneity related to the presence of Glc*p* instead of Glc*p*N, as was observed previously in the core oligosaccharide of *P. shigelloides* O33:H3 (Table S1 and Figure S3). The OSIII structure is also identical to the core oligosaccharide identified in the *P. shigelloides* O51 (strain CNCTC 110/92) [31]. The structural identity of the core oligosaccharide was further confirmed serologically. In the immunoblotting analysis the antiserum specific for the OS of *P. shigelloides* O51 reacted vividly with the fast migrating bands of the SDS-PAGE-separated LPS fractions composed of the core oligosaccharide linked to lipid A. Reactions were also observed for the LPS fractions composed of the lipid A-core oligosaccharides substituted by the increasing number of the *O-*repeats (Figure S4).

### 2.3. Structure Analysis of the O-Specific Polysaccharide

**Figure 3:**
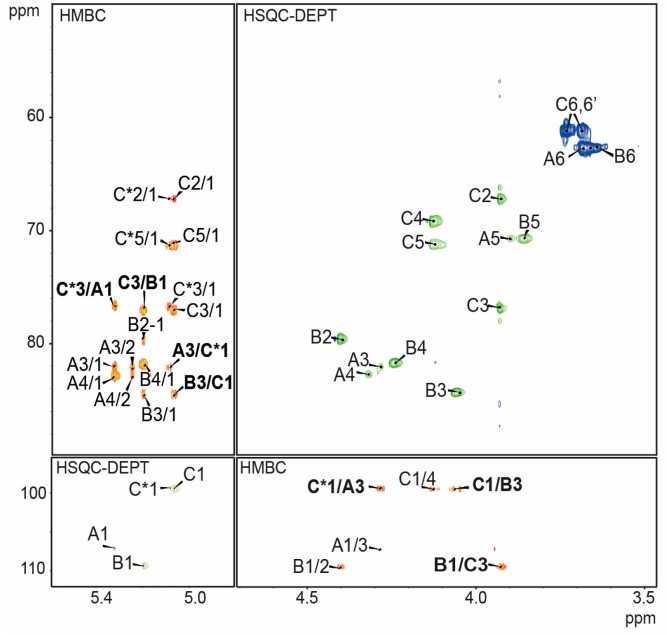
Selected ^1^J_H,C_- and ^3^J_H,C_-connectivities in HSQC-DEPT and HMBC spectra of the isolated PSI of *Plesiomonas shigelloides* 78/89 LPS. The inter-residue connectivities from the anomeric atoms in HMBC spectra are marked in bold. The spectra were obtained for ^2^H_2_O solutions at 600 MHz and 30 °C. The uppercase letters refer to sugar residues in the *O-*specific polysaccharide, as described in Table 1.

Initial analysis of the *P. shigelloides* 78/89 bacteria and LPS using HR-MAS NMR spectroscopy provided a structural fingerprint of the *O-*antigen chemotype, including the ^1^H and ^13^C chemical shift values for the *O-*specific polysaccharide in situ. Composition analysis of the PSI polysaccharide fraction together with determination of the absolute configuration confirmed the presence of d-galactose residues in galacto-furanose and galacto-pyranose ring forms. The NMR spectra of the isolated PSI contained main signals for three anomeric protons and a distinct signal (δ_H_ 2.11 ppm) in the region of acetyl groups. The spin systems for each residue (denoted as uppercase letters through the entire text, tables, and figures) were assigned using COSY, TOCSY, HSQC-DEPT, HSQC-TOCSY, HMBC, and NOESY NMR experiments (Table 1 and Figure 3).

The coupling patterns of the identified spin systems in the COSY and TOCSY spectra indicated the *galacto*-configuration of all residues.

Residue **A** (δ_H1_/δ_C1_ 5.32/107.1 ppm) was identified as a 3-substituted 2-*O*-acetyl-β-d-galactofuranose (β-d-Gal*f*2OAc) on the basis of the high chemical shift values of anomeric carbon (δ 107.1), C-2 (δ 81.8), C-3 (δ 82.1) and C-4 (δ 82.9) as well as the similarities of the chemical shift values of H-2, H-3, and H-4 to the published data [32]. This was further supported by the observed correlation of H-1 with both C-3 and C-4, and a lack of correlation with C-5 in the HMBC spectrum (Figure 3). The characteristic large downfield shift of the H-2 signal (δ_H_ 5.25) and its multiple bond correlation with a carbonyl carbon resonance at δ_C_ 173.1 ppm in the HMBC spectrum indicated an ester substitution at this position by an *O-*acetyl group (δ_H_/δ_C_ 2.11/20.5). Similarly, residue **B** (δ_H_/δ_C_ 5.2/109.4 ppm, ^1^J_C1,H1_ ~171 Hz) was recognized as the 3-substituted-β-d-galactofuranose devoid of the *O-*acetyl. The chemical shift values except the H-2 were similar to these of residue A. The relatively high chemical shift of the C-3 signal (δ 84.5) indicated a substitution by another residue. Residue **C** with the H1/C1 signal at δ_H_/δ_C_ 5.07/99.5 ppm, ^1^J_C1,H1_ ~175 Hz was assigned as a 3-substituted α-d-galactopyranose (α-d-Gal*p*) based on the large vicinal couplings between H-2 and H-3, the small vicinal coupling constants between H-3, H-4 and H-5, as well as relatively high chemical shift value of the C-3 signal (δ 76.9). The heteronuclear multiple bond correlations between H-1 and both C-3 and C-5 observed in the HMBC spectrum confirmed the pyranose ring size. The presence of the C* variant (δ_H_/δ_C_ 5.08/99.4 ppm) of residue **C** reflected the changes of chemical shifts induced by the *O-*acetylation of the disaccharide repeating unit and indicated that two forms of the disaccharide repeating units exist in the *O-*specific polysaccharide: the *O-*acetylated A-C* and non-*O*-acetylated B-C.

The minor anomeric signal (δ_H_/δ_C_ 5.11/95.4 ppm, residue C’) and the corresponding spin system observed at a lower contour level in the 2D spectra of the PSI showed chemical shift values similar to the unsubstitited α-d-galactopyranose [33] and was attributed to a non-reducing terminal variant of residue **C**, defining the biological repeating unit of the *O-*specific polysaccharide.

The NOESY and HMBC (Table 2, Figure 3) spectra showed inter-residue cross-peaks between the transglycosidic protons, and between the anomeric protons and carbons at the linkage position as well as carbons and the protons at the linkage position, respectively. The inter-residue NOEs were identified between H-1 of residue **A** and H-3 of residue C* and between H-1 of residue C* and H-3 of residue **A** for the *O-*acetylated RU as well as between H-1 of residue **B** and H-3 of residue **C**, and between H-1 of residue **C** and H-3 of residue **B** for the non-acetylated form of the repeating unit.

The combined data indicated the disaccharide repeating unit of *P. shigelloides* 78/89 *O-*PS with the following structure: 
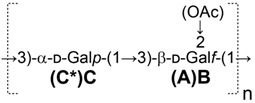


*O*-deacetylation of the *O-*polysaccharide rendered only signals for two anomeric protons of residues **B** and **C** in the spectrum. Thus, the PSI NMR profile reverted to that of the d-galactan I, and the NMR spectrum became identical to the HR-MAS NMR spectrum of *K. pneumoniae* Kp20 LPS (Figure S5).

The *O-*acetylation of the *O-*specific polysaccharide in the PSI fraction was not complete. The degree of the *O-*acetylation was determined by integration of the anomeric signal of the 2-*O*-Ac-Gal*f* residue (δ_H_ 5.32 ppm) in the ^1^H NMR spectrum of PSI relative to the resolved resonance of an anomeric proton at 5.20 ppm (integral value of 1, corresponding to a single proton). The ratios of the integral values have shown that ~32% of the repeating units were *O-*acetylated in the PSI, generating the *O-*acetylation-related heterogeneity. This level of substitution was sufficient to disguise the chemotype of pure d-galactan I. Interestingly, the NMR spectra also indicated that there was no *O-*acetylation of the repeating units in the OSII fraction (Figure S2). As the isolation procedures can cleave off or induce migration of the *O-*acetyl groups to neighboring positions we have also compared volumetric integration values for the H-1/C-1 resonances in the HR-MAS HSQC-DEPT NMR spectra in situ. The relative content of the *O-*acetyl groups was calculated as 29.9% in the isolated *O-*specific polysaccharides, 29.4% in the isolated LPS and 25.7% in the LPS directly on bacterial cells.

### 2.4. Serological Analysis

**Figure 4:**
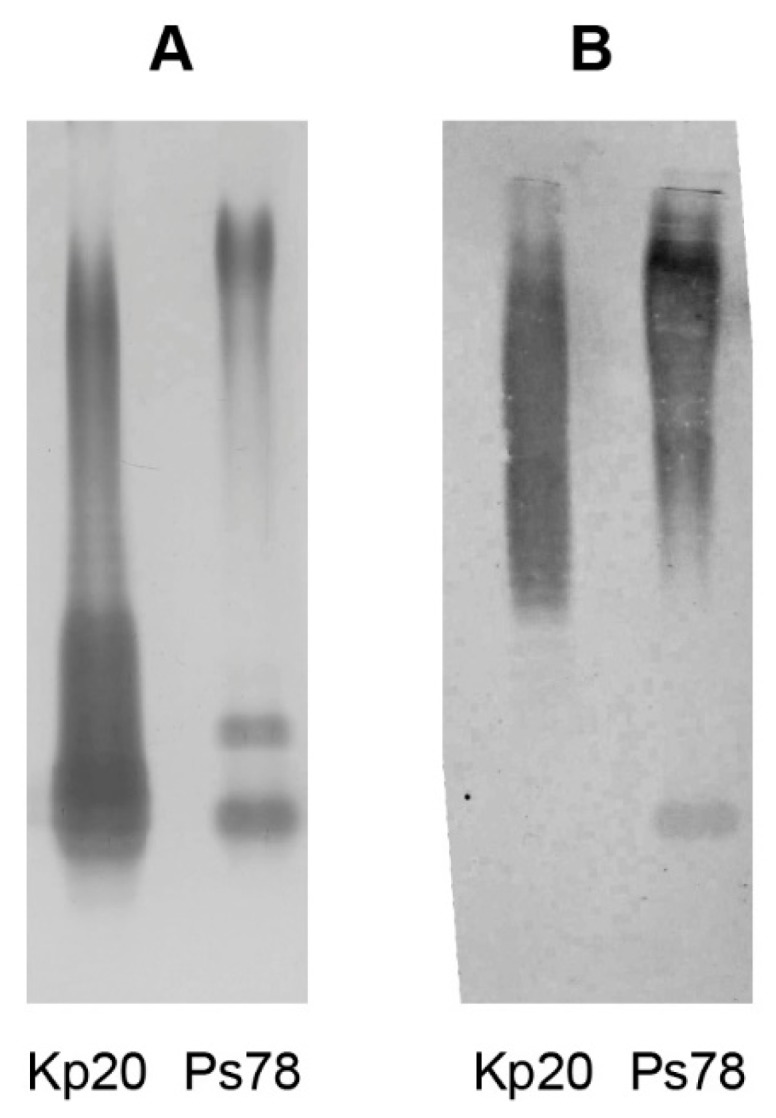
SDS-PAGE analysis and reactivities of *P. shigelloides* serotype O12-specific polyclonal antibodies with LPSs of *P. shigelloides* 78/89 and *K. pneumoniae* Kp20 in immunoblotting. LPSs of *P. shigelloides* 78/89 (Ps78) and *K. pneumoniae* Kp20 (Kp20) were analyzed by SDS-PAGE (1.25 μg/lane) using 15% separating gel and visualized by silver staining method (**A**) or transblotted onto nitrocellulose (**B**). Polyclonal antibodies specific for the O12 serotype were 200-fold diluted.

Reactivities of the serotype O12-specific serum with the homologous *P. shigelloides* 78/89 and *K. pneumoniae* Kp20 LPSs were investigated by immunoblotting, ELISA experiments and ELISA inhibition assays. The silver stained SDS-PAGE of *P. shigelloides* strain 78/89 LPS showed both low and high molecular mass LPS bands (Figure 4A). In the immunoblotting analysis (Figure 4B) the antiserum against *P. shigelloides* O12 reacted vividly with the high molecular mass bands of the homologous *O-*antigen of *P. shigelloides* 78/89.

**Figure 5:**
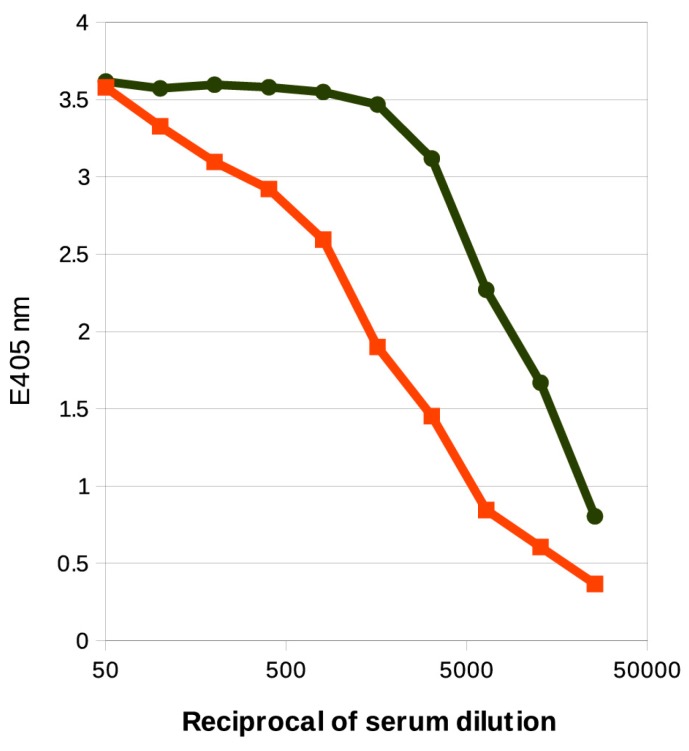
ELISA profiles of polyclonal antibodies specific for the *P. shigelloides* serotype O12 with LPSs of *P. shigelloides* 78/89 (black line) and *K. pneumoniae* Kp20 (red line) as solid-phase antigens (10 μg/mL). The depicted ELISA values are the means of four replicates.

We also observed a cross-reaction of the anti-*P. shigelloides* O12 serum with the high molecular mass bands of *K. pneumoniae* Kp20 LPS. However, this reaction covered a broader range of the *O-*specific chain length. In ELISA assay both lipopolysaccharides reacted with the serum specific for the serotype O12, although the profiles differed (Figure 5).

This cross-reaction indicates the presence of antibodies that recognize the common structural element—the non-*O*-acetylated disaccharide repeating unit segments in the *O-*antigens of *P. shigelloides* 78/89 and *K. pneumoniae* Kp20. To further assess the role of the *O-*acetylation in the recognition by antibodies we used ELISA inhibition assay. The inhibitory effects of the *O-*deacetylated *O-*specific polysaccharide of *P. shigelloides* strain 78/89 on the reactivity of the anti-*P. shigelloides* O12 serum with LPS of *P. shigelloides* strain 78/89 and *K. pneumoniae* Kp20 were compared. The *O-*specific polysaccharide devoid of the *O-*acetyl groups inhibited the reactions of the anti-*P. shigelloides* O12 antibodies with LPS of *K. pneumoniae* Kp20 to a maximal value of ~84% at the inhibitor concentration of 62.5 µg/mL, indicating the presence of antibodies specific for the epitopes different from the pure d-galactan I. The reaction of anti-*P. shigelloides* O12 antibodies with LPS of *P. shigelloides* strain 78/89 was inhibited by the *O-*deacetylated *O-*PS to a maximal value of ~94% at the inhibitor concentration of 250 µg/mL (Figure S6). Thus the combined data from ELISA and ELISA inhibition tests indicate the presence of a pool of antibodies specific for the d-galactan I in the anti-*P. shigelloides* O12 serum, but more importantly, the analysis suggests that the *O-*acetylation creates additional *O-*serotype-related antigenic determinants within this *O-*antigen that are recognized by the O12-specific serum. However, the exact contribution of the epitopes comprising the *O-*acetyl groups could not be determined as neither the linear nor spatial distribution of the *O-*acetyls in the PSI was known.

## 3. Discussion

The *O-*acetylation of the *O-*antigens is typically investigated using the isolated *O-*specific polysaccharides, obtained by mild acidic hydrolysis of the ketosidic linkage between the Kdo residue of the heteropolysaccharide and lipid A. This isolation procedure may result in partial cleavage of the *O-*acetyl groups and their migration to neighboring positions. Non-stoichiometric *O-*acetylation of the *O-*specific polysaccharides, both native and introduced by the isolation methods, makes the NMR spectra more complex, alters the signals of structure reporter groups for known chemotypes and introduces new ones. Here we have presented HR-MAS NMR as a technique that allows for a comparison of the degree of *O-*acetylation of the *O-*antigens at different stages of the isolation procedure, as the changes in the HR-MAS NMR spectra provide immediate distinction of varying structures [18]. This approach to the structural analysis of the *O-*antigens revealed that the mere presence of the *O-*acetyl groups can mask the dominant chemotype. The structure of *P. shigelloides* strain 78/89 LPS is unique among *Plesiomonas* strains. However, the pattern in the HR-MAS spectrum of this LPS appeared similar to the HR-MAS profile of *K. pneumoniae* Kp20 LPS. The latter is built of a disaccharide repeating unit →3)-β-d-Gal*f*-(1→3)-α-d-Gal*p*-(1→ which is identical to the non-*O*-acetylated repeating unit variant of *P. shigelloides* strain 78/89 LPS.

The use of NMR spectroscopy, MALDI-TOF mass spectrometry, and immunochemical analysis provided detailed structural data on the *O-*antigen of *P. shigelloides* strain 78/89 LPS. The *O-*specific polysaccharide isolated from the LPS by conventional methods showed a pattern typical for smooth-type enterobacterial lipopolysaccharide. The identified disaccharide →3)-α-d-Gal*p*-(1→3)-β-d-Gal*f*2OAc-(1→ unit exhibits *O-*acetylation-related heterogeneity and constitutes a new chemotype among *O-*antigens of the *P. shigelloides* strains. As indicated by the HR-MAS NMR analysis in situ and on the isolated LPS, the relative degree of the *O-*acetylation and the position of the *O-*acetyl group was consistent throughout the different stages of the preparations and analysis. The serological analysis confirmed that the strain represents serotype O12 [14]. Thus the structure of *P. shigelloides* serotype O12 LPS has now been established.

We have also observed that the *O-*specific polysaccharide repeating units were nearly identical to these of *K. pneumoniae* Kp20 LPS [34]. However, it is worth noting that their *O-*specific chain length differed and that the *O-*antigen of *P. shigelloides* 78/89 showed higher-degree of polymerization, as indicated by SDS-PAGE and immunoblotting analysis.

The shared structural element in the *O-*specific polysaccharide that occurs in those two strains also explains the observed cross-reactivity with the antiserum specific for the *O-*antigen of *P. shigelloides* O12. Since only 32% of β-d-Gal*f* molecules in *P. shigelloides* 78/89 *O-*PS are *O-*acetylated, it is no surprise that a pool of antibodies in the anti-*P. shigelloides* O12 serum recognizes the d-galactan I of *K.pneumoniae* and the *O-*acetylation effects the cross-reactivity of the strains by creating an additional epitope recognized by the O12-specific antibodies. The role of nonstoichiometric substitutions on O antigenicity is commonly acknowledged. It is a well-known phenomenon among *Salmonella* and *Shigella* as the introduction of acetyl or glucose can form new *O-*types [10].

The β-d-galactofuranosyl-(1→3)-d-galactopyranose known as d-galactan I [35] is present in the LPS of the genus *Klebsiella*. This disaccharide is considered a valuable model for studies of biochemical pathways for formation of the Gal*f*-contaning glycans present in the bacterial cell wall complex (extracellular glycocalyx) [36]. These glycans may play a critical role in the survival and pathogenicity of microorganisms; therefore, their biosynthetic pathways could be attractive targets for drugs that act by inhibiting cell wall biosynthesis [36]. *P. shigelloides* 78/89 expresses LPS with the modified d-galactan I structure of the *O-*antigen.

*Klebsiella* and *Plesiomonas* are Gram-negative organisms that are frequently co-isolated from intestinal infections. They are also isolated from different clinical specimens including respiratory tract. Their common residence and a niche-specific selection may have contributed to the fact that the strains share epitopes. The cross-reactivities of *P. shigelloides O-*antigens with antisera raised against some strains of the genus *Shigella* have been previously reported [15,28]. These strains of *Plesiomonas* and *Shigella* either had some common epitopes or structures of their *O-*antigens were identical. The structural determinant →3)-α-d-Gal*p*-(1→3)-β-d-Gal*f*-(1→ described here for *P. shigelloides* 78/89 LPS is a part of the O1 antigen [35], which is the most clinically prevalent serotype of *K. pneumoniae*. The type-specific cross-reactivities among non-closely related bacteria are a subject of special interest, particularly in the case of antibiotic-resistant pathogens such as *Klebsiella*. Furthermore, it was reported that *K. pneumoniae* strains that belong to O1 and O2 (termed d-galactan I) serogroups cause over 50% of all *Klebsiella* infections worldwide [37].

Typically, *O-*serotypes of *Klebsiella* and *Plesiomonas* were classified using cross-absorbed sera, indicating the differences among the epitopes recognized by polyclonal antibodies with no immediate correlation to the chemotypes of their *O-*antigens.

The *O-*antigens are appealing targets for active and passive immunizations with a potential for therapeutic applications. However, it is important to uncover subtle structural attributes of LPS molecules that may have impact on the desired specificity of antibodies [38]. It is worth noting that all such structural features could be identified using HR-MAS NMR data alone. The results of our study demonstrate a potential application of the HR-MAS NMR technique for screening of the bacterial *O-*antigen structures in situ, providing robust information on the chemotype of the *O-*antigens.

## 4. Materials and Methods

### 4.1. Bacteria

*P. shigelloides* strain CNCTC 78/89, classified as serovar O12:H12 according to Aldova’s antigenic scheme [14], was obtained from the Institute of Hygiene and Epidemiology, Prague, Czech Republic. The bacteria were grown on a Davies medium enriched with glucose for 24 h in 37 °C, killed with 0.5% phenol, and harvested as described previously, yielding 6.48 g of freeze-dried bacteria. *K. pneumoniae* strain 20 (Kp20, serogroup O2) is a bloodstream clinical isolate and was kindly provided by Szilvia Melegh (Department of Medical Microbiology and Immunology, Medical School, University of Pecs, Pecs, Hungary) [34]. Samples of freeze-dried bacteria and the isolated LPS used for the HR-MAS analysis were generously supplied by Jolanta Lukasiewicz (Hirszfeld Institute of Immunology and Experimental Therapy, Wroclaw, Poland).

### 4.2. Lipopolysaccharides and O-Specific Polysaccharide Fractions

LPS was isolated from bacterial cells by the hot phenol/water extraction [39] and purified by ultracentrifugation [40]. Subsequently, *O-*specific polysaccharide was isolated by mild acid hydrolysis (1.5% acetic acid containing 2% SDS at 100 °C for 15 min) of LPS (50 mg). SDS was removed by extraction with 96% ethanol and the pellet was suspended in water and centrifuged. The supernatant was fractionated by gel permeation chromatography, on Bio-Gel P-10 (1.6 × 100 cm) equilibrated with 0.05 M pyridine/acetic acid buffer, pH 5.6. The chromatography yielded a main fraction containing *O-*specific polysaccharide (PSI, 7 mg), separated from shorter *O-*specific polysaccharide chains linked to the core (OSII, <<1 mg) and two fractions containing unsubstituted core oligosaccharides (OSIII, 1.2 mg and OSIV, 5.4 mg). The eluates were monitored with a Knauer differential refractometer and all fractions were checked by NMR spectroscopy MALDI-TOF mass spectrometry (MS).

### 4.3. Analytical Procedures

Monosaccharides were analyzed as their alditol acetates by GC-MS. Methylation was performed on the isolated PS according to the method described by Hakomori [41]. Alditol acetates and partially methylated alditol acetates were analyzed by GC-MS using an ITQ 700 (Thermo Scientific, Waltham, MA, USA) system, equipped with a HP-1 fused-silica capillary column (0.2 mm × 12.5 m) and a temperature gradient from 150 to 270 °C at 12 °C·min^−1^. The absolute configurations of the sugars were determined as described by Gerwig et al. [42] using (−)-2-butanol for the formation of 2-butyl glycosides. The trimethylsilylated butyl glycosides were then identified by comparison with authentic samples on GC-MS. 

### 4.4. O-Deacetylation of Polysaccharide

Polysaccharide (5 mg) was treated with aqueous 12.5% NH_3_ (1 mL) at room temperature for 16 h, after which the solution was diluted with water and freeze-dried. The product was analyzed by NMR spectroscopy.

### 4.5. Partial Acid Hydrolysis

The polysaccharide (0.5 mg) was used for hydrolysis with 0.5 M trifluoroacetic acid (1 mL) at 80 °C. A sample (20 µL) was taken every 30 min for 2 h and the progress of hydrolysis was checked by MALDI-TOF MS.

### 4.6. SDS-PAGE and Serological Analysis

The LPS was analyzed by SDS-PAGE according to the method of Laemmli with modifications [43]. The LPS bands were visualized by the silver staining method [44] and by immunoblot using polyclonal rabbit antisera specific for the *O-*antigen of *P. shigelloides* O12 and for the OS of *P. shigelloides* O33 in separate experiments. Immunoblotting was done as previously described [40]. An ELISA, using LPS as the solid-phase antigen, was performed by a modification [45] of the method described by Voller et al. [46]. The detection systems consisted of a goat anti-rabbit IgG conjugated with alkaline phosphatase (Bio-Rad, Richmond, CA, USA) as a second antibody and 5-bromo-4-chloro-3-indolylphosphate-nitroblue tetrazolium and p-nitrophenylphosphate for immunoblotting and the ELISA test, respectively. ELISA inhibition test was performed as described in [47]. Briefly, the serum (100 µL) at a concentration twice as high as the one giving E_405nm_ value in the range (0.8–2.2) was mixed with 100 µL of inhibitor solution (inhibitor concentration range from 250 µg/mL down to 0.49 µg/mL) and incubated for 1 h at 37 °C. The mixture (100 µL) was then transferred to a microtiter plate with LPS, incubated for 15 min with shaking and followed by the subsequent steps of standard ELISA protocol.

### 4.7. Mass Spectrometry

MALDI-TOF MS spectra of the PSI, the partially hydrolyzed PSI and the OS fractions were acquired using Bruker Autoflex III (Bruker Daltonics, Bremen, Germany) time-of-flight instrument. Spectra were recorded in positive and negative modes. 2,5-Dihydroxybenzoic acid was used as matrix.

### 4.8. NMR Spectroscopy

NMR spectra of bacteria (~4 mg dry mass) and LPS (~3 mg) suspensions in ^2^H_2_O (total volume of ~30 µL in the Bruker Kel-F inserts) were obtained using the HR-MAS technique on a Bruker Avance III 600 MHz spectrometer (Bruker BioSpin, Rheinstetten, Germany). HR-MAS NMR experiments were carried out at a spin rate of 4 kHz at 27 °C (the actual temperature of the bearing gas) using a Bruker 4 mm HR-MAS probe and a ZrO_2_ rotor, as previously described [48]. NMR spectra of the isolated polysaccharide and oligosaccharides were recorded for ^2^H_2_O solutions at 30 °C on Bruker Avance III 600 MHz spectrometer using a 5 mm inverse detection QCI cryoprobe and 1.7 mm TXI microprobe. The polysaccharide and oligosaccharide fractions were repeatedly exchanged with ^2^H_2_O, with intermediate lyophilization. The data were acquired and processed using Topspin 3.1 (Bruker BioSpin, Rheinstetten, Germany). The processed spectra were assigned with the help of the SPARKY program [49]. The signals were assigned by one- and two-dimensional experiments (COSY, TOCSY, NOESY, HMBC, HSQC-DEPT, and HSQC-TOCSY). In the TOCSY experiments the mixing times used were 30, 60, and 100 ms. The delay time in the HMBC experiment was 60 ms and the mixing time in the NOESY experiment was 200 ms. Regions of interest (ROI) in the HR-MAS HSQC-DEPT spectra were visualized using the rNMR program [50].

The relative content of the *O-*acetyl groups in the *O-*specific polysaccharides was determined by integration of the relevant signals from their 1D ^1^H NMR spectra acquired with a 30° pulse and a relaxation delay of 1 s. Volumetric integration of the selected resonances in the HSQC-DEPT spectra was performed by the Gaussian fit method using built-in functions of the SPARKY program.

## Figures and Tables

**Table 1:** ^1^H and ^13^C NMR chemical shifts of the *O-*specific polysaccharide of *P. shigelloides* strain CNCTC 78/89 ^a^.

Residue	Chemical Shifts (ppm)					
	H-1	H-2	H-3	H-4	H-5	H-6, H-6′
	C-1	C-2	C-3	C-4	C-5	C-6
**A** →3)-β-d-Gal*f*2OAc-(1→	5.32	5.25 ^b^	4.28	4.32	3.89	3.68, 3.65
	107.1	81.8	82.1	82.9	70.8	62.7
**B** →3)-β-d-Gal*f*-(1→	5.20	4.40	4.06	4.24	3.86	3.66, 3.68
	109.4	79.8	84.5	81.8	70.8	62.7
**C** →3)-α-d-Gal*p*-(1→	5.07	3.93	3.93	4.12	4.12	3.73, 3.68
	99.5	67.2	76.9	69.2	71.3	61.1
**C′** α-d-Gal*p*-(1→ ^c^	5.11	3.90	4.01	4.12	4.27	3.8, 3.75
	95.4	68.5	69.6	70.3	71.2	60.7
C* →3)-α-d-Gal*p*-(1→	5.08	3.94	3.95	4.11	4.11	3.69, 3.62
	99.4	67.2	76.7	69.1	71.3	60.9

^a^ Spectra were obtained for ^2^H_2_O solutions at 30 °C. Acetone was used as internal reference (δ_H_/δ_C_ 2.225/31.05 ppm); ^b^ The *O-*2 of this residue is *O-*acetylated (δ_CH3CO_ 2.11/20.5 ppm, 173.1 ppm); ^c^ The non-reducing terminal variant of residue C.

**Table 2:** Selected inter-residue NOE and ^3^J_H,C_-connectivities from the anomeric atoms of the *O-*antigen unit of PS *P. shigelloides* strain CNCTC 78/89.

Residue	Atom H-1/C-1	Connectivities to		Inter-Residue
	(ppm)	δ_C_	δ_H_	Atom/Residue
**A** →3)-β-d-Gal*f*2OAc-(1→	5.32/107.1	76.7	3.95	C-3, H-3 of C*
**B** →3)-β-d-Gal*f*-(1→	5.20/109.4	76.9	3.93	C-3, H-3 of **C**
**C** →3)-α-d-Gal*p*-(1→	5.07/99.5	84.5	4.06	C-3, H-3 of **B**
C* →3)-α-d-Gal*p*-(1→	5.08/99.4	82.1	4.28	C-3, H-3 of **A**

## Supplementary Materials

Supplementary materials can be found at www.mdpi.com/1422-0067/18/12/2572/s1.

## Abbreviations

LPS	Lipopolysaccharide
PS	O-specific polysaccharide
OS	Oligosaccharide
HR-MAS	High-Resolution Magic Angle Spinning
NMR	Nuclear Magnetic Resonance
COSY	Correlation spectroscopy
TOCSY	Total correlation spectroscopy
NOESY	Nuclear Overhouser Effect spectroscopy
HSQC	Heteronuclear Single Quantum Coherence
DEPT	Distortionless Enhancement by polarization transfer
HMBC	Heteronuclear Multiple Bond Correlation
GC	Gas chromatography
MS	Mass spectrometry
MALDI-TOF	Matrix-assisted laser desorption ionization time-of-flight
SDS	Sodium dodecyl sulfate
