# Supplementary material for: Structural Masquerade of Plesiomonas shigelloides Strain CNCTC 78/89 O-Antigen—High-Resolution Magic Angle Spinning NMR Reveals the Modified d-galactan I of Klebsiella pneumoniae
Source: Int J Mol Sci. 2017 Nov 29;18(12):2572. doi: 10.3390/ijms18122572 (PMC5751175; doi:10.3390/ijms18122572)
Supplement: Supplementary file 1 [file ijms-18-02572-s001.pdf]

Structural Masquerade of *Plesiomonas shigelloides* Strain CNCTC 78/89 *O*-Antigen—High-Resolution Magic Angle Spinning NMR Reveals the Modified D-galactan I of *Klebsiella pneumoniae*

Karolina Ucieklak, Sabina Koj, Damian Pawelczyk and Tomasz Niedziela *

Hirszfeld Institute of Immunology and Experimental Therapy, Wroclaw, Poland; karolina.ucieklak@iitd.pan.wroc.pl (K.U.); sabina.koj@iitd.pan.wroc.pl (S.K.); damianpawelczyk@yahoo.pl (D.P.)

* Correspondence: tomasz.niedziela@iitd.pan.wroc.pl; Tel.: +48-71-337-1172

The supplementary data and raw NMR spectra:

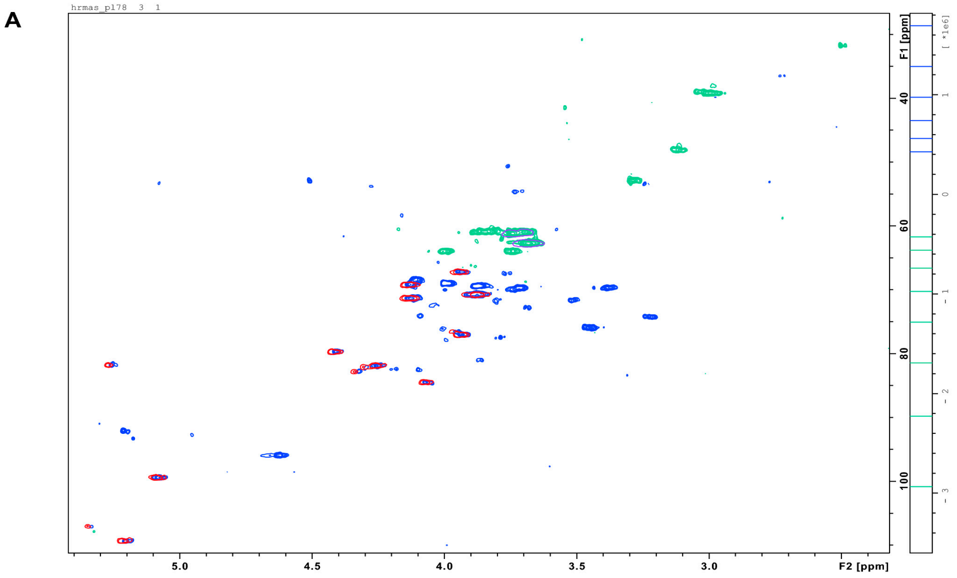

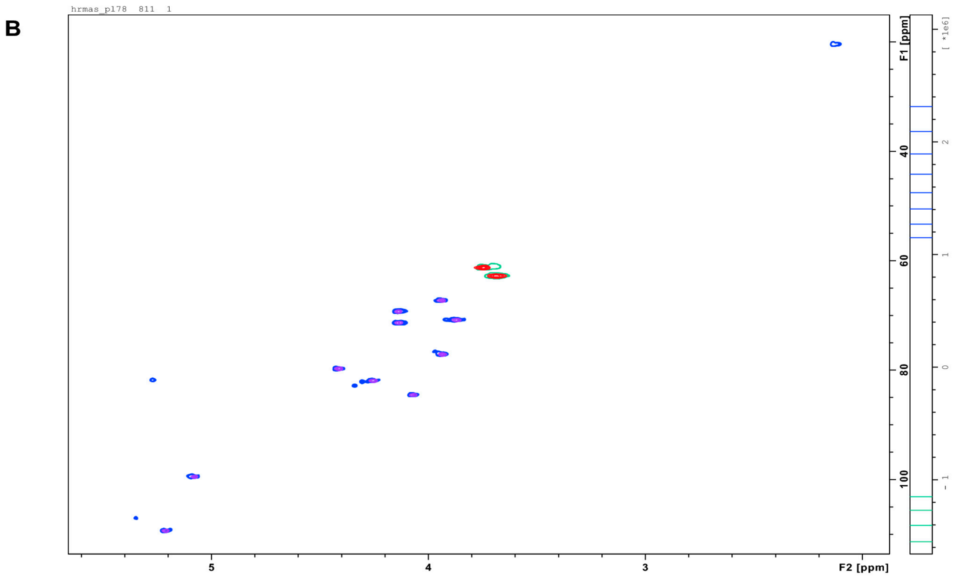

Figure S1. (**A**) HR-MAS HSQC-DEPT NMR spectra of the O-antigen of *P. shigelloides* strain CNCTC 78/89 acquired directly on bacteria and on the isolated LPS (overlay spectrum); (**B**) HR-MAS HSQC-DEPT NMR spectra of the isolated LPS of *P. shigelloides* strain CNCTC 78/89 and the D-galactan I of *K. pneumoniae* Kp20 LPS (overlay spectrum).

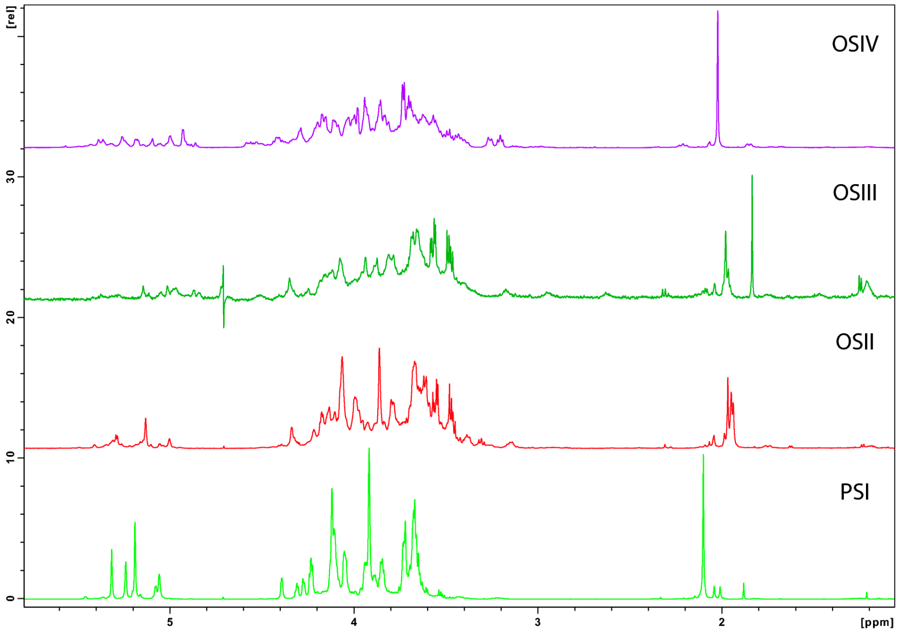

Figure S2. 1D NMR spectra of the fractions identified as the O-specific polysaccharide (PSI), a fraction composed of short O-specific chains substituted by core oligosaccharide (OSII), and the core oligosaccharide (OSIII and OSIV).

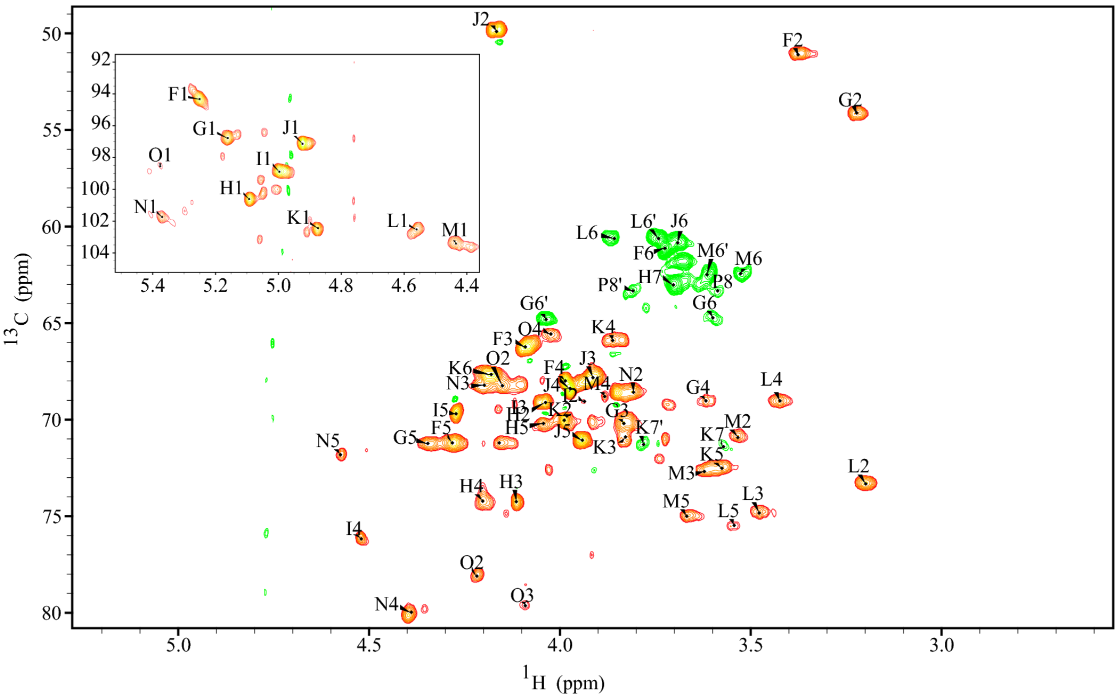


```
                                F                 I                K              M
                              α-GalN-(1→4)-α-GalA-(1→7)-α-Hep       β-Gal
                                                                ↓               ↓
            J               G              N                    7 O             4 H           P
R-4)-α-GalNAc-(1→6)-α-GlcN-(1→4)-α-GalA-(1→3)-α-Hep-(1→3)-α-Hep-(1→5)-Kdo
                                                                 2
                                                                 ↑ L
                                                               β-Glc
```

Figure S3. HSQC-DEPT spectrum of the isolated core oligosaccharide (fraction OSIV) of *P. shigelloides* 78/89 LPS. The inset shows the region of H-1,C-1 anomeric resonances. The uppercase letters refer to sugar residues in the oligosaccharide, as described in Table S1.

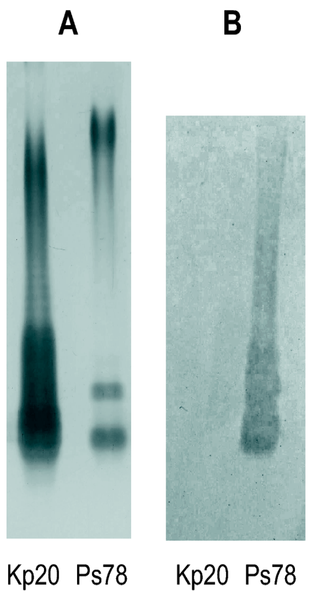

Figure S4. Reactivities of polyclonal antibodies specific to the core oligosaccharide of *P. shigelloides* serotype O51 (strain CNCTC 110/92) with the LPS of *P. shigelloides* 78/89 and *K. pneumoniae* Kp20 in immunoblotting (**B**). For reference the SDS-PAGE analysis depicted in Figure 4A of the main text is reproduced alongside (**A**). The polyclonal antibodies were raised against the OS-BSA neoglycoconjugate of the core oligosaccharide of *P. shigelloides* strain CNCTC 110/92.

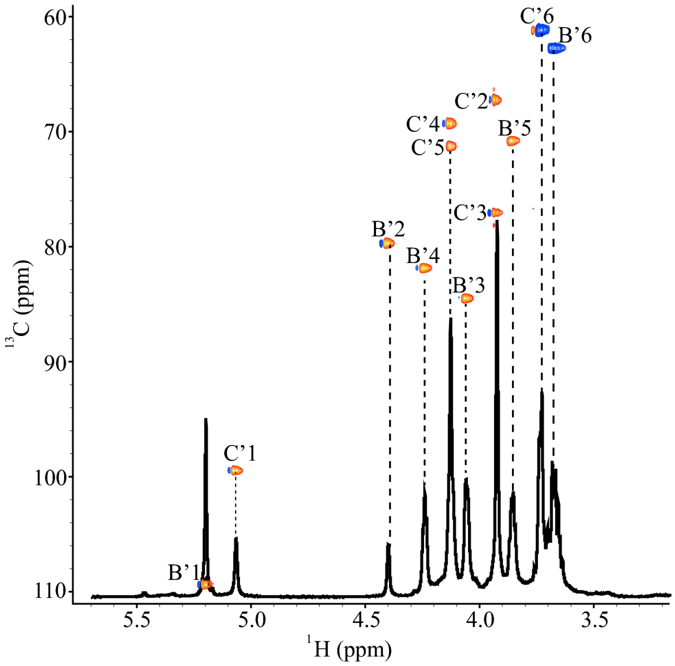

Figure S5. HSQC-DEPT NMR spectrum of the O-deacetylated O-specific polysaccharide of *P. shigelloides* strain CNCTC 78/89. The overlay depicts the 1D ^{1}H NMR profile. The primed-uppercase letters correspond to sugar residues, as described for the non-O-acetylated form of PSI.

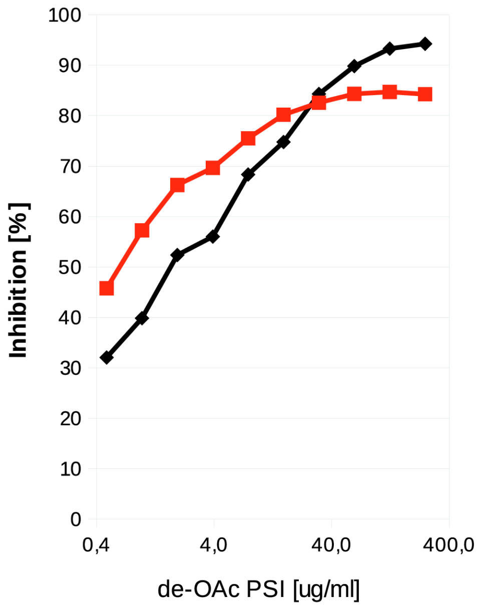

Figure S6. Inhibition of anti-O12 specific antibodies reaction with LPS of *P. shigelloides* 78/89 (black line) and *K. pneumoniae* Kp20 (red line) as solid-phase antigens (10 μg/ml) by O-deacetylated PSI fraction of *P. shigelloides* 78/89. The depicted inhibition values were calculated from the means of four replicates in the ELISA inhibition assay. The reference $E_{405\ nm}$-values obtained with no inhibitor were 2.189 (Ps78) and 0.701 (Kp20).

Table S1. ^{1}H and ^{13}C chemical shifts of the core oligosaccharide fraction (OSIV) of *P. shigelloides* strain CNCTC 78/89[a]

Residue		Chemical shifts [ppm]							
		H-1	H-2	H-3	H-4	H-5	H-6, 6'	H-7	H-8
		C-1	C-2	C-3	C-4	C-5	C-6	C-7 (CH_3CO)	C-8
**F**	α-D-Gal*p*N-(1→	5.25	3.38	4.09	3.99	4.28	3.73		
		94.3	51.1	66.2	68.0	71.2	61.1		
**G**	→6)-α-D-Glc*p*N-(1→	5.16	3.22	3.83	3.62	4.35	3.60, 4.04		
		96.9	54.2	70.2	69.0	71.2	64.8		
**H**	→3,4)-L-α-D-Hep*p*-(1→	5.09	4.05	4.11	4.20	4.16	nd[b]	3.70	
		100.6	70.2	74.3	74.2	71.2	nd	63.0	
**I**	→4)-α-D-Gal*p*A-(1→	4.99	3.94	4.04	4.52	4.27			
		98.9	69.1	69.1	76.2	69.7	175.9		
**J**	α-D-Gal*p*NAc-(1→	4.92	4.17	3.91	3.97	3.94	3.69	(2.03)	
		97.1	49.9	67.8	68.4	71.1	60.9	(21.9, 174.7)	
**K**	→7)-L-α-D-Hep*p*-(1→	4.87	3.99	3.83	3.86	3.58	4.18	3.57, 3.78	
		102.5	69.8	70.9	65.9	72.5	67.7	71.3	
**L**	β-D-Glc*p*-(1→	4.56	3.20	3.48	3.42	3.54	3.86, 3.74		
		102.5	73.3	74.8	69.0	75.5	60.6		
**M**	β-D-Gal*p*-(1→	4.44	3.54	3.62	3.88	3.67	3.53, 3.62		
		103.4	70.9	72.7	68.8	75.0	62.5		
**N**	→4)-α-D-Gal*p*A-(1→	5.37	3.81	4.20	4.39	4.57			
		101.7	68.6	68.2	80.0	71.8	175.3		
**O**	→2,3,7)-L-α-D-Hep*p*-(1→	5.38	4.18	4.09	4.02	nd	nd		
		98.6	73.2	79.6	65.6	nd	nd		
**P**	→5)Kdo	-	-	1.82, 2.14	4.08	4.11	3.83[c]	nd	3.80, 3.58
		nd	96.7	34.0	66.0[c]	74.3[c]	71.0[c]	nd	63.4

[a]Spectra were obtained for $^{2}H_2O$ solutions at 30 °C. Acetone was used as internal reference (δ_H/δ_C 2.225/31.05 ppm). The chemical shifts are the average values obtained from the set of complementary experiments (COSY, TOCSY, HSQC-DEPT, HSQC-TOCSY and HMBC); [b]Not determined; [c]The assignments of the signals are tentative as the Kdo spin system was not fully resolved.
